# A Rare Manifestation of Sunburn Rash: A Case Report of Brachioradial Pruritus

**DOI:** 10.7759/cureus.82806

**Published:** 2025-04-22

**Authors:** Oladoyin Ogunbayo Jolaoye

**Affiliations:** 1 Internal Medicine - Pediatrics, Order of Saint Francis Medical Center, University of Illinois College of Medicine at Peoria, Peoria, USA

**Keywords:** brachioradial, brachioradial pruritus, hyperpigmentation sunburn and skin aging, rash, sunburn

## Abstract

Brachioradial pruritus (BRP) is a type of localized neuropathic dysesthesia of the dorsolateral upper extremities that is commonly found in fair-skinned females. It is worsened by exposure to bright sunlight or ultraviolet radiation (UVR), a subtype of neuropathic pruritus that is delayed and difficult to diagnose. It typically involves a combination of C5-C6 dermatomal distribution of the dorsolateral arm (often bilateral), spinal abnormality, and radiculopathy. The ice-pack sign is universally known to be associated with BRP.

We present a case of a 41-year-old Caucasian female who presented with complaints of sunburn to bilateral arms, pins and needles, and extreme itching sensation in the bilateral upper arms. The patient tried lotions, cortisone cream, and oral Benadryl without relief. The patient reported that the rash worsened with heat and improved with ice packs. On the primary survey, vitals were stable, but the presence of dark erythema on the bilateral upper arms was visible. The patient was recommended to apply silver sulfadiazine 1% cream twice daily, oral gabapentin 300 mg twice daily, and topical diclofenac gel as needed. The patient saw a primary care physician (PCP), dermatology, and neurology, with broad differential diagnoses, and underwent several treatments for other diseases, but the primary diagnosis of BRP was not established.

Early recognition and diagnosis of BRP, by taking a thorough history and physical exam, and utilization of the ice-pack test, is critical for prompt management and avoiding unnecessary workup.

## Introduction

Brachioradial pruritus (BRP) is a type of localized neuropathic dysesthesia of the dorsolateral upper extremities that also includes postherpetic neuralgia, multiple sclerosis, and notalgia paresthetica [1,2]. It is also considered a subtype of neuropathic pruritus that is commonly found in fair-skinned females in about 70% of cases, and is difficult to diagnose [1,2]. BRP is worsened by exposure to bright sunlight or ultraviolet radiation (UVR), and typically involves a combination of C5-C6 dermatomal distribution of the dorsolateral arm involvement (bilateral in 75% of cases), spinal abnormality, and radiculopathy [1,2]. The cause of BRP is unknown, but it has been attributed to solar radiation present in the summer [1,2]. The ice-pack sign is universally known to be associated with BRP. It is a test that involves placing an ice-pack on the affected region, with immediate improvement of pruritus, but the pruritus returns once the ice-pack is removed [2].

## Case presentation

**Figure 1:**
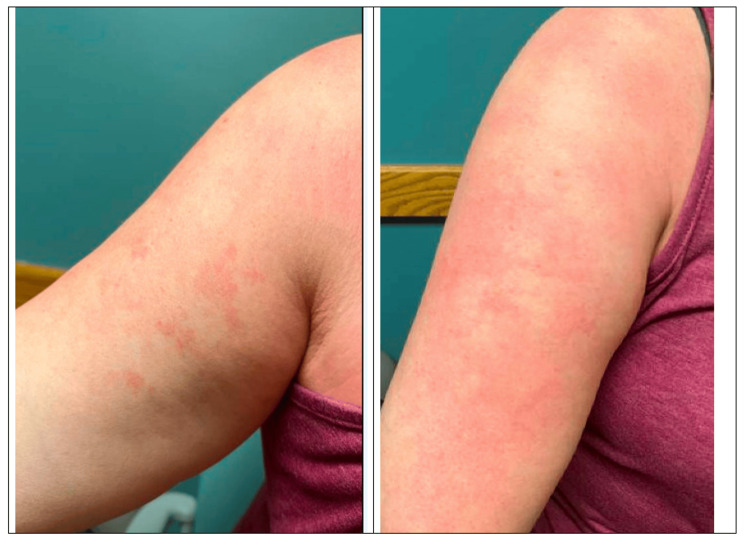
Maculopapular rash in the right upper extremity involving the C5 dermatome

A 41-year-old Caucasian female with a past medical history of generalized anxiety disorder (GAD) and sunburns presented to her primary care physician's (PCP) office with complaints of sunburn to bilateral arms, pins and needles, and extreme itching sensation in the bilateral upper arms. The patient decided on a PCP visit after failed therapy with lotions, over-the-counter (OTC) cortisone cream, and oral Benadryl. The patient reported that the rash worsened with heat and improved with ice. She also endorsed changes in color to the rash, hot to the touch, tender, mild swelling, and significant nighttime awakening related to the pain associated with the rash. On the primary survey, vitals were stable, but the presence of dark erythema on the bilateral upper arms and an atypical skin rash were visible on physical examination, as seen in Figure 1. The patient was recommended to apply silver sulfadiazine 1% cream twice daily for the atypical skin rash, take gabapentin 300 mg twice daily by mouth for neuropathic pain, and apply diclofenac gel for inflammatory pain as needed, and a referral was sent to dermatology.

The dermatologist performed a punch biopsy and an allergy test that showed a nickel allergy. The biopsy report showed sparse lymphohistiocytic, superficial, perivascular inflammatory cell infiltrates; features were non-specific, with suggestions that it may be seen in some drug or viral exanthems; gyrate erythema; urticaria; tumid lupus erythematosus; pigmented purpuric dermatosis; and post-inflammatory pigmentary alteration. The patient was given a diagnosis of pruritus versus erythromelgia and advised to start topical gabapentin 6%, ketamine 4%, lidocaine 3% cream to apply to the affected area three times a day, and referred to neurology for further evaluation.

**Figure 2:**
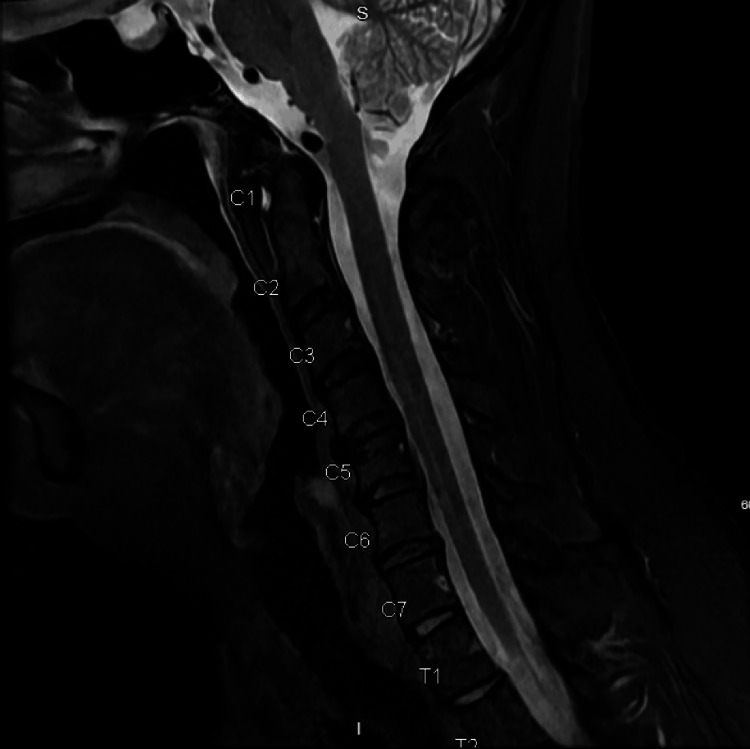
MRI C-spine with and without contrast showing normal diameter of central spinal canal of the cervical spine MRI, magnetic resonance imaging

The neurologist recommended magnetic resonance imaging (MRI) of the cervical spine with and without contrast, which was normal, as seen in Figure 2. The patient was diagnosed with abnormal skin changes and sensory disturbance of the right lateral upper arm with C5 dermatomal distribution, with recommendations to have dermatology refill topical pain medication (gabapentin 6% - ketamine 4% - lidocaine 3% cream), and have the PCP titrate up the gabapentin dose.

The patient returned to the PCP for follow-up, where she was diagnosed with BRP through a process of exclusion. Her oral gabapentin dose was increased to 300 mg three times daily, and she was continued on the topical cream prescribed by dermatology, with significant improvement of the skin rash and associated pain within one month. 

## Discussion

The BRP etiology is unknown [3]. It has been attributed to be caused by prolonged solar radiation exposure in the summer months, exacerbating underlying cervical spine issues, and is associated with a cervical nerve root injury in the setting of osteoarthritis or trauma, resulting in the compression of the nerve root [3,4]. In a study, 7 out of 10 patients with notalgia paresthetica (a sensory nerve entrapment syndrome involving the posterior rami of the T2-T6 nerve roots) had cervical and/or thoracic radiographic changes, along with dermatomal localization of the itching [2,4]. Though our patient did not present with trauma or a history of osteoarthritis, she presented with neuropathic pain and paresthesias in the bilateral forearms. Multiple studies have found that BRP has been associated with cervical radiculopathy in about 57% of patients [4]. 

The itch and rash associated with BRP are mostly located on the dorsolateral aspect of sun-exposed forearms and are known to be exacerbated by sun exposure during the summer months [2,3]. The pruritus can sometimes extend across the back and chest, and has been linked to nervous system pathology, especially as a consequence of cervical nerve disease [3,4]. It has been hypothesized that UVR damage to the nerve endings may also lead to neuropathic BRP in individuals predisposed to cervical spine disease [5]. Most patients describe the itch as picking, prickling, burning, or plain itch involving the unilateral or bilateral dorsolateral forearms, which are neuropathic features, hinting that BRP can be associated with a neurogenic origin [2,3,5]. Our patient described her itch as a pins-and-needles-like sensation, exacerbated by exposure to the sun on her bilateral forearms.

Patients with BRP report that the application of ice packs provides relief from the itch. This is called the ice-pack sign, which is pathognomonic for BRP [6]. The ice pack offers temporary relief from the itch. Our patient reported that the itch worsened on exposure to sunlight and was relieved when ice packs were placed on her forearms bilaterally.

It is believed that both cervical spine disease and sun-induced cutaneous nerve injury are major contributors to BRP in patients [2-4]. Treatment for BRP has been difficult, but some reported treatments include oral gabapentin, topical capsaicin, oral carbamazepine, cervical spine manipulation, neck traction, physiotherapy, anti-inflammatory medications, surgical resection of cervical ribs, and sunlight avoidance [3]. Our patient noted significant improvement within one month with oral gabapentin and topical gabapentin 6%, ketamine 4%, lidocaine 3% cream.

## Conclusions

Early recognition and diagnosis of BRP by taking a thorough history and physical exam, and the utilization of the ice-pack test, are critical for prompt management and avoiding unnecessary workup. A patient presenting with sunburn to the bilateral arms, pins-and-needle sensation, extreme itching sensation in the bilateral upper arms, neuropathic dysesthesia of the dorsolateral upper extremities, a visible rash, and stating improvement of pruritus with cold compresses should consider BRP as a top differential.
